# Metabolomic Profiles and Pathways in Osteoarthritic Human Cartilage: A Comparative Analysis with Healthy Cartilage

**DOI:** 10.3390/metabo14040183

**Published:** 2024-03-25

**Authors:** Hope D. Welhaven, Avery H. Welfley, Priyanka Brahmachary, Annika R. Bergstrom, Eden Houske, Matthew Glimm, Brian Bothner, Alyssa K. Hahn, Ronald K. June

**Affiliations:** 1 Department of Chemistry & Biochemistry, Montana State University, Bozeman, MT 59717, USA; 2 Department of Mechanical & Industrial Engineering, Montana State University, Bozeman, MT 59717, USA; 3 Department of Chemical & Biological Engineering, Villanova University, Villanova, PA 19085, USA; 4 Department of Biological and Environmental Sciences, Carroll College, Helena, MT 59625, USA

**Keywords:** osteoarthritis, cartilage, metabolomics, mass spectrometry

## Abstract

Osteoarthritis (OA) is a chronic joint disease with heterogenous metabolic pathology. To gain insight into OA-related metabolism, metabolite extracts from healthy (n = 11) and end-stage osteoarthritic cartilage (n = 35) were analyzed using liquid chromatography–mass spectrometry metabolomic profiling. Specific metabolites and metabolic pathways, including lipid and amino acid pathways, were differentially regulated in osteoarthritis-derived and healthy cartilage. The detected alterations in amino acids and lipids highlighted key differences in bioenergetic resources, matrix homeostasis, and mitochondrial alterations in OA-derived cartilage compared to healthy cartilage. Moreover, the metabolomic profiles of osteoarthritic cartilage separated into four distinct endotypes, highlighting the heterogenous nature of OA metabolism and the diverse landscape within the joint in patients. The results of this study demonstrate that human cartilage has distinct metabolomic profiles in healthy and end-stage OA patients. By taking a comprehensive approach to assess metabolic differences between healthy and osteoarthritic cartilage and within osteoarthritic cartilage alone, several metabolic pathways with distinct regulation patterns were detected. Additional investigation may lead to the identification of metabolites that may serve as valuable indicators of disease status or potential therapeutic targets.

## 1. Introduction

Osteoarthritis (OA) is the leading cause of disability worldwide. Since 1999, the number of global cases has increased by an astonishing 113%, equating to ~528 million individuals affected in 2019 [1,2]. In the United States alone, 32.5 million adults have OA, costing USD 185 billion annually [3,4,5,6]. At the heart of OA’s insidious progression lies the gradual breakdown of articular cartilage (AC) and other joint tissues. The imbalanced activity between matrix anabolism and catabolism contributes to the observed changes in AC, other tissues, and fluids affected by OA (i.e., underlying bone, synovium, synovial fluid). Previous studies examined altered metabolism in various OA-associated tissues and their cell types, such as chondrocytes, to investigate disease-associated metabolic activity [7,8,9]. However, significant limitations of many studies are that they were performed in vitro and/or lacked healthy human controls, thereby hindering a complete understanding of the role metabolism plays in OA development. 

Moreover, the complex nature of OA can manifest differently between individuals. Specifically, symptom severity, rate of progression, response to treatment, pain perception as well as other factors can vary from person to person [10,11,12]. Therefore, a “one-size-fits-all” approach to the treatment and prevention of OA is limited. More recent studies describe OA as a group of symptoms encompassing multiple distinct phenotypes and endotypes rather than a single disease [13]. Previously, phenotype was defined as a single or collection of disease characteristics that explain differences between patients and their outcomes, such as symptom severity [11,12,13]. Conversely, endotype is defined functionally and pathologically by a molecular mechanism noting that different mechanisms can lead to the same manifestation, such as end-stage OA [14]. Examining OA phenotypes and endotypes may shed light on the epidemiological origins and development of OA, unveil biomarkers, and lead to targeted interventions for sub-populations of OA individuals, all of which have potential to improve patient outcomes. 

Metabolomics, the study of small molecule intermediates called metabolites [15], is advantageous for generating and investigating OA metabolic endotypes because it detects thousands of metabolites. This enables the generation of biochemical signatures that represent the overall physiological state of the tissue. To our knowledge, two prior studies used a similar approach to examine synovial fluid metabolism from OA individuals. Here, researchers characterized different regulation patterns, or endotypes, based on detected differences in biochemical signatures between healthy and OA individuals [16,17]. However, this same approach has yet to be applied to osteoarthritic cartilage. To begin filling these gaps in knowledge, we compared the metabolome of radiography-confirmed end-stage OA cartilage (Kellgran–Lawrence grades III and IV) with that of healthy cartilage using liquid chromatography–mass spectrometry (LC-MS) metabolomics.

Thus, the primary objective of this study was to identify disease-associated OA metabolomic profiles to shed light on the pathological mechanisms underlying OA. The secondary objective was to examine and classify endotypes of OA. Furthermore, we used tandem LC-MS (LC-MS/MS) for the biochemical identification of key metabolites. This has the potential to identify novel biomarkers and drug targets to slow, halt, or reverse OA progression. With this approach, we aimed to uncover specific metabolic endotypes and metabolite identities to serve as potential indicators of disease status or therapeutic intervention across sub-populations of OA individuals.

## 2. Materials and Methods

### 2.1. Articular Cartilage Sample Obtainment

Under IRB approval, 35 femoral heads from end-stage OA patients were obtained following total joint arthroplasty from local musculoskeletal clinics. Partial patient information including age, sex, and BMI was provided (Supplemental Table S1). However, radiographic scans were not obtained due to the IRB approval only permitting partial patient information to be shared. Post-mortem cartilage samples were obtained from donors without joint disease (articular engineering) to serve as healthy controls for comparison.

### 2.2. Metabolite Extraction and Mass Spectrometry Analysis

The cartilage samples were shaved from the femoral head prior to metabolite extraction. All cartilage samples (n = 35 OA, n = 11 healthy) were extracted using a previously established protocol [18]. All cartilage samples were weighed prior to extraction to normalize metabolite intensity off cartilage weight. Notably, the weights of healthy cartilage were consistently measured (100 mg), while the weights of OA cartilage were variable, as they were obtained from end-stage OA patients, each of whom had different amounts of intact cartilage (minimum = 16.8 mg, maximum = 223.3 mg, average = 73.0 mg). Next, the cartilage shavings were submerged in 3:1 methanol/water and homogenized using a tissue homogenizer (SPEX Sample Prep 1200 GenoLyte, Fisher Scientific, Metuchen, NJ, USA). Homogenization included 15 cycles of 20 s and resting periods of 2 min. Next, the samples were briefly vortexed and stored at −20 °C overnight to promote protein precipitation. The following day, the samples were vortexed again and centrifuged for 10 min at 16,100× *g* at 4 °C, and the supernatants were collected and dried via vacuum concentration.

The dried supernatants were then resuspended with 1:1 acetonitrile/water, stored at −20 °C for 30 min, and then centrifuged again for 10 min at 16,100× *g* at 4 °C. Similarly, the supernatants were dried via vacuum concentration and then prepared for liquid chromatography–mass spectrometry (LC-MS) by resuspending with 1:1 acetonitrile/water. Additionally, 4 pooled samples (n = 1 healthy, n = 3 OA) were generated for identification purposes. For the healthy pool, aliquots of 5 µL from each healthy extract were combined. For the OA pools, 3 pooled samples were generated in the same way, combining aliquots of 5 µL from 10 randomly selected OA extracts per individual pool.

The extracted cartilage, both healthy and OA, underwent mass spectrometry analysis as previously described [19]. In brief, an Aquity UPLC Plus interfaced through an electrospray ionization source to a Waters Synapt XS was used. A Cogent Diamond Hydride HILIC column (150 × 2.1 mm) at a flow rate of 0.400 µL/min was used to separate the metabolites in 19 min over an elution gradient (A = 95/5% water/acetonitrile, B = 95/5% acetonitrile/water). Every 10 injections, blank samples containing mass spectrometry-grade water were injected to minimize the spectral drift and assess the LC-MS performance. The cartilage extracts and the blank samples underwent standard LC-MS, whereas the pooled samples underwent liquid chromatography–tandem mass spectrometry (LC-MS/MS) at a constant high-energy ramp of 30–50 V for secondary ionization to derive metabolite identifications. All samples—including cartilage extracts, pools, and blanks—were ran at the same time consecutively.

### 2.3. Statistical and Metabolomic Profiling

The LC-MS data, consisting of mass-to-charge ratios (m/z), relative metabolite abundance, and retention time, were processed using MSConvert [20] and XCMS [21]. Prior to data analysis, the metabolites associated with each cartilage sample were normalized by the pre-extraction-recorded cartilage shaving weight. Previously established analysis pipelines were used [19,22] and executed in MetaboAnalyst [23], where the data underwent an integrity check to remove noise and avoid overfitting, interquartile range normalization, log transformation, and autoscaling (mean-centered/standard deviation of each metabolite feature). In brief, hierarchical clustering analysis (HCA), principal component analysis (PCA), and partial least-squares discriminant analysis (PLS-DA) were used to visualize dissimilarities in the metabolomic profiles between healthy and OA cartilage, as well as examine OA endotypes. *T*-test, fold change, and volcano plots were used to assess the significance and magnitude of changes. Moreover, these populations of metabolite features were differentially regulated between the groups, and those identified by these tests underwent pathway enrichment analysis using the MetaboAnalyst’s Functional Analysis feature, which utilizes the *mummichog* algorithm to predict networks of functional activity from metabolite features of interest. The pathway library Human MFN was used as the primary reference library to match metabolite features to putatively identified metabolites (mass tolerance: 5 parts per million (ppm); positive mode, version 1). Significance for pathway analyses and all other statistical tests was determined using a false discovery rate (FDR)-corrected significance level of *p* < 0.05.

### 2.4. Metabolite Identification

A major hurdle in LC-MS-based metabolomics is metabolite identification [24]. To address this challenge, the pooled samples were subjected to LC-MS/MS involving fragmentation, allowing for the analysis of parent and daughter fragment ions. These data were manually analyzed to confirm the metabolite identifications as follows. Firstly, all LC-MS/MS data from the pooled samples were imported, peak-picked, and aligned using Progenesis QI (Nonlinear Dynamics, Newcastle, UK, version 3.0). The utilization of Progenesis improves the efficiency of identification and uses a computational framework that allows for the exploration of thousands of putative metabolite identifications across various databases. Here, the Human Metabolome Database (HMDB) [25] was utilized to compare the theoretical fragmentation patterns to the acquired fragmentation patterns of parent and daughter ions. For a metabolite identity to be deemed valid and subsequently investigated manually, we required it to receive a fragmentation score and overall progenesis score greater than 12 and 60 out of 100, respectively. These score criteria were based on mass error, isotope distribution, and retention time. Once the identified metabolites were narrowed based on these set scores and parameters, they were matched against populations of LC-MS-based metabolite features distinguished by statistical analyses comparing OA and healthy cartilage, as well as OA endotypes. To minimize false identifications, an error threshold of 10 ppm between observed and Progenesis-identified metabolites was enforced.

## 3. Results

### 3.1. Global Metabolomic Profiles of Osteoarthritis and Healthy Cartilage Unveil Altered Cellular Mechanisms Associated with Disease

**Figure 1:**
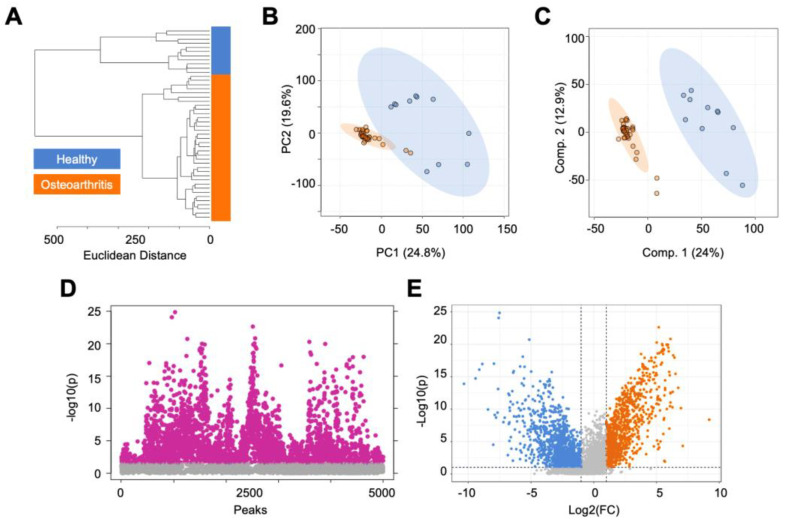
The metabolomic profiles of human cartilage from healthy and osteoarthritis patients are metabolically distinct. (**A**) Hierarchical clustering analysis found that healthy and osteoarthritic cartilage samples cluster separated from each other. (**B**) Principal component analysis, an unsupervised test, found a minimal overlap of principal components 1 and 2, accounting for 44.4% of the variability in the dataset. (**C**) Partial least-squares discriminant analysis, a supervised test, showed complete separation of healthy and diseased cartilage samples, with components 1 and 2 accounting for 36.9% of the variability in the dataset. (**D**) *T*-test analysis detected 2842 metabolite features, with a false discovery rate-adjusted *p*-value less than 0.05. (**E**) Volcano plot analysis, using fold change and statistical significance, distinguished differentially regulated metabolites between healthy and diseased cartilage. Specifically, 1010 metabolite features were more abundant in diseased cartilage compared to healthy cartilage (log2(FC) > 2, *p* < 0.05), whereas 1399 were more abundant in healthy cartilage compared to diseased cartilage (log2(FC) < −2, *p* < 0.05). Orange = osteoarthritis. Blue = healthy.

In total, 10,853 metabolite features were detected by LC-MS across all cartilage samples. To visualize and assess metabolomic differences between healthy and OA cartilage, we used unsupervised (HCA, PCA) and supervised (PLS-DA) multivariate tests. HCA, visualized by a dendrogram and measured using Euclidean distance, displayed the clear separation of healthy and OA cartilage (Figure 1A). A similar trend was observed when using PCA and PLS-DA, where a near-perfect separation of the groups was displayed, demonstrating metabolomic profiles reflective of the disease status of the cartilage (Figure 1B,C).

Next, *t*-test and volcano plot analyses were performed to distinguish dysregulated populations of metabolite features between healthy and OA cartilage. The populations distinguished by both analyses were then analyzed using the MetaboAnalyst’s Functional Analysis feature to find biological pathways that differed in regulation across the groups. Volcano plot analysis found 1010 metabolite features that were more abundant in OA cartilage compared to healthy cartilage (Figure 1D). These metabolite features mapped to numerous lipid-related pathways (omega-3 and -6 fatty acid metabolism, fatty acid activation and oxidation, polyunsaturated and saturated fatty acid beta-oxidation, glycosphingolipid metabolism), the carnitine shuttle, leukotriene metabolism, and others (Table 1, Supplemental Table S2). Conversely, volcano plot analysis also found 1399 metabolite features that were more abundant in healthy cartilage compared to OA cartilage (Figure 1D). These features mapped to the urea cycle, purine metabolism, glycerophospholipid metabolism, vitamin metabolism (K, E), squalene and cholesterol biosynthesis, aminosugar metabolism, and various amino acid metabolic pathways (methionine, cysteine, histidine, glycine, serine, alanine, threonine, tryptophan) (Table 1, Supplemental Table S2). The t-test distinguished 2842 metabolite features that were significantly dysregulated between the groups (FDR *p* < 0.05) (Figure 1E).

Additionally, features distinguished by volcano plot analysis (Supplemental Table S3) and *t*-test (Supplemental Table S4) were matched to putative identifications made using LC-MS/MS to unveil metabolic indicators of disease. Putatively identified metabolites that were statistically significant in both *t*-test and volcano plot analyses and were more abundant in healthy cartilage compared to OA cartilage included N-acetyl-leukotriene E4, demethylphylloquinone, 7C-aglycone, androsterone sulfate, and others (Supplemental Figure S1A). The majority of the identified metabolites distinguished by these analyses were more abundant in healthy cartilage, with the exception of guanidinoethyl methyl phosphate, cervonyl carnitine, erythromycin propionate, and glycocholic acid (Supplemental Figure S1B). Collectively, these findings unveiled specific metabolites and metabolic pathways that showed altered cellular mechanisms in OA and reflect the disease status of cartilage.

### 3.2. Endotype Characterization Supports the Heterogenous Nature of Osteoarthritis

**Figure 2:**
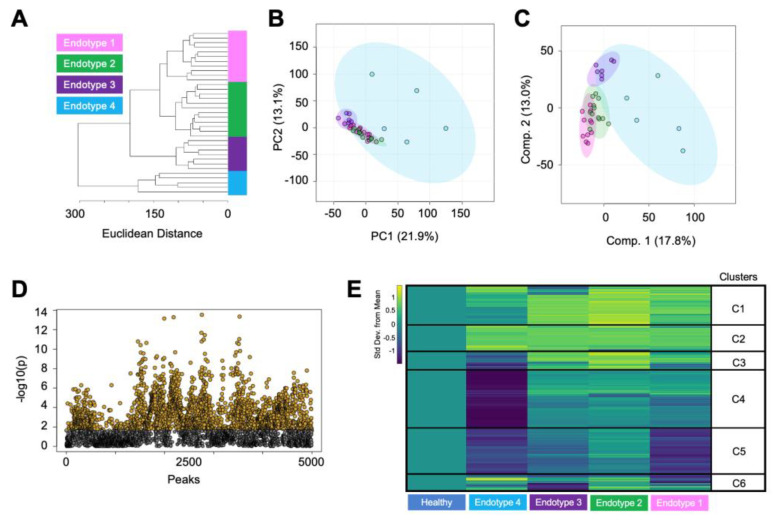
Metabolomic assessment of osteoarthritic cartilage classifies unique patient endotypes. (**A**) Hierarchical clustering of cartilage from patients with osteoarthritis showing that they clustered into 4 distinct endotypes. (**B**) These endotypes were further examined and visualized by principal component analysis. Principal components 1 and 2 accounted for 35% of the variability in the dataset and showed a moderate overlap of the osteoarthritis endotypes. (**C**) Partial least-squares discriminant analysis slightly refined the separation of the groups with components 1 and 2, accounting for 30.8% of the variability in the dataset. (**D**) ANOVA analysis detected 2506 metabolite features with a false discovery rate-adjusted *p*-value less than 0.05. (**E**) Metabolite features distinguished by ANOVA were visualized using a median metabolite intensity heatmap, where the osteoarthritis endotypes were normalized to healthy cartilage. Heatmap clusters of co-regulated metabolite features (C1–C6) that were differentially regulated across the OA endotypes were then subjected to pathway analyses to pinpoint distinct metabolomic endotypes across osteoarthritic cartilage. Columns represent endotype groups, and rows indicate metabolite features. Warmer colors (yellow) indicate higher metabolite abundance, whereas cooler colors (blue) indicate lower metabolite abundance. Endotype colors correspond to endotypes as follows: pink—endotype 1; green—endotype 2; purple—endotype 3; blue—endotype 4.

To examine the heterogenous nature of OA metabolism and better understand differences in the diverse metabolic landscape within OA, we examined metabolomic endotypes. Clustering techniques—HCA (Figure 2A) and ensemble clustering (Supplemental Figure S2)—were utilized to identify OA endotypes across all cartilage samples. The application of both methods aimed to minimize subjectivity in delineating OA cartilage endotypes and determine OA participants that consistently clustered together. These analyses unveiled four distinct endotypes in the OA participants. Considering patient-specific factors like age, the ratio of males to females, and the overall number of participants within each endotypes, we found no clear pattern related to participant demographics that correlated with these four endotypes (Supplemental Table S1).

Once the endotypes were distinguished and patient-specific factors were examined, we used PCA and PLS-DA to gain additional insight into these endotypes. This revealed a limited overlap between the endotype groups (Figure 2B,C). Notably, endotype 4 exhibited considerable variability, portrayed by a substantial ellipse. In contrast, endotypes 1–3 showed a closer metabolomic resemblance, where proximity of smaller and tighter clustered ellipses was observed. The subsequent ANOVA analysis identified 2506 metabolite features that were significantly different between endotypes, with FDR-corrected *p* < 0.05 (Figure 2D). This subset of features was then matched to putative identifications made using the LC-MS/MS data. The identifications consisted of lipid and lipid-like metabolites including cervonyl carnitine, lucidenic acid A, 6-epi-7-isocucurbic acid glucoside, various phosphatidylcholine species, and others (Supplemental Figure S3A, Supplemental Table S5). Additionally, metabolites related to arachidonic acid and leukotriene metabolism were identified, including arachidonic acid, panaxydol linoleate, leukotriene F4, and N-acetyl-leukotriene E4 (Supplemental Figure S3B, Supplemental Table S5).

Additionally, this subset of statistically significant metabolite features was further examined using median-metabolite-intensity heatmap analysis normalized to healthy cartilage to find dissimilarities in metabolomic regulation across the four OA endotype groups compared to healthy cartilage (Figure 2E). For this analysis, the median intensity for each metabolite feature was calculated, and this same calculation was then extended to each endotype group to observe and compare major differences in metabolomic regulation between endotypes. Metabolite features within heatmap clusters 1 and 2, which exhibited higher abundances across endotypes compared to healthy cartilage, mapped to 16 statistically significant pathways including leukotriene metabolism, selenoamino acid metabolism, the carnitine shuttle, and numerous lipid-related pathways (Table 2, Supplemental Table S6). Heatmap clusters 3 and 4, comprising metabolites whose levels were lowest in endotype 4 and highest in other endotypes, mapped to 16 statistically significant pathways including vitamin A metabolism, phytanic acid peroxisomal oxidation, lysine and tyrosine metabolism, keratan sulfate degradation, N-glycan degradation, lineolate metabolism, butanoate metabolism, and various lipid-related pathways. Metabolites composing heatmap cluster 5 showed relatively lower levels across endotypes compared to healthy samples and mapped to seven statistically significant pathways including purine metabolism, leukotriene metabolism, urea cycle, aminosugar metabolism, and various amino acid pathways (methionine, cysteine, tryptophan, aspartate, asparagine). Lastly, heatmap cluster 6 consisted of metabolites with mixed regulation patterns across endotypes; however, no statistically significant pathways were detected. All pathways reported had an FDR-corrected *p* < 0.05. Collectively, these findings underscore the heterogenous nature of OA metabolism among patients with OA and provide compelling evidence to support the diverse landscape of metabolic regulation associated with this disease.

## 4. Discussion

While altered metabolism is increasingly recognized as a crucial factor in the development of OA, further data are needed to understand the role of aberrant metabolism in OA pathophysiology. This study found distinct human cartilage-derived metabolomic profiles in healthy and end-stage OA patients. Through a comprehensive analysis aimed at discerning differences in the metabolome of healthy and OA cartilage, we found that several metabolites and pathways associated with matrix metabolism, lipid metabolism, mitochondrial function, vitamin metabolism, and amino acid metabolism were differentially regulated in healthy and OA cartilage. Moreover, investigation of metabolic diversity within the metabolome of OA cartilage alone mapped to distinct metabolomic endotypes, displaying the heterogeneous nature of OA. Considering these metabolomic findings, a greater understanding of altered cartilage metabolism in OA may lead to the identification of candidate biomarkers and drug targets to slow, halt, or reverse cartilage damage in end-stage OA.

### 4.1. Matrix Metabolism

OA cartilage exhibited greater evidence of altered matrix metabolism compared to healthy cartilage. Specifically, keratan sulfate degradation and N-glycan degradation were upregulated in OA cartilage compared to healthy cartilage. Keratan sulfate, a type of glycosaminoglycan (GAG), plays a vital role in cartilage matrix homeostasis and maintenance. The homeostatic GAG content in both synovial fluid (SF) and cartilage is indicative of joint health, whereas an increase in GAGs within the SF suggests increased cartilage turnover. This is subsequently reflected by a decrease in GAG content within the cartilage itself [26,27,28]. Furthermore, alterations in N-glycan degradation likely reflect changes in joint lubrication, as N-glycans are an important component of lubricin, a glycoprotein that lines cartilage surfaces and serves as a key joint lubricant with chondroprotective properties [29]. 

### 4.2. Lipid and Mitochondria-Related Metabolism

Several lipid-related pathways were upregulated in OA cartilage compared to healthy cartilage and were differentially regulated across OA endotypes. Notably, the present study identified several significant lipid-related pathways that were previously linked to OA, including the carnitine shuttle, arachidonic acid metabolism, omega-3 and -6 metabolism, glycosphingolipid metabolism, and glycerophospholipid metabolism. Cartilage relies on bone and SF for lipid transport, underscoring the critical role of lipid metabolism in maintaining cartilage homeostasis. Arachidonic acid (AA), leukotriene F4, N-acetyl-leukotriene E4, and panaxydol linoleate were identified using the LC-MS/MS data, were present at higher concentrations in OA cartilage compared to healthy cartilage, and differed in abundance across the OA endotypes (Supplemental Figures S1 and S3). AA, a type of omega-6 polyunsaturated fatty acid known to be associated with inflammation, is typically found at lower levels in healthy cartilage, and its level increases as OA progresses [30]. Additionally, elevated AA levels were detected in OA SF [16,31] and synovium [32], and more broadly, the severity of synovitis and histological changes in OA were correlated with the serum levels of omega-3 and -6 [33,34].

The detection of perturbed lipid pathways and a handful of identified lipid species in OA cartilage may reflect adaptive responses in mitochondrial function and biofuel utilization in response to OA. While healthy cartilage relies on both glucose and lipids as energy sources, OA cartilage exhibits a greater dependence on lipids [35,36]. This metabolic switch to lipid utilization can lead to the accumulation of lipids, the increased production of reactive oxygen species and nitric oxide, and decreased ATP production, leading to eventual tissue breakdown and death [37,38,39,40].

Central to this metabolic switch is the carnitine shuttle, which plays a key role in regulating the oxidative status by transporting lipids across the mitochondrial membrane to generate ATP. The upregulation of the carnitine shuttle in OA cartilage compared to healthy cartilage and across OA endotypes is supported by previous studies which detected not only the carnitine shuttle but also elevated levels of acylcarnitine and other carnitine species [17] in the SF of OA patients. Moreover, cervonyl carnitine, a type of acylcarnitine, was identified using the LC-MS/MS data; its levels were significantly higher in all OA cartilage samples compared to healthy cartilage and differed across the OA endotypes (Supplemental Figures S1 and S3). Cervonyl carnitine is often produced as a result of a disorder or disease (i.e., cancer, diabetes, cardiovascular disease) and disrupts energy production [41]. It has been well documented that OA perturbs energy production in cartilage; therefore, the detection of this species in the present study could be a result of receiving cartilage from donors with radiography-confirmed OA.

We previously detected cervonyl carnitine in SF from patients who sustained a traumatic knee injury [19]. Here, we hypothesized that a metabolic switch toward lipid utilization and the involvement of mechanisms like the carnitine shuttle were necessary to meet the heightened energy demands post-injury and that ongoing analysis of these species may help manage post-traumatic OA. Thus, the detection of cervonyl carnitine in OA cartilage and in SF post-injury further highlights its potential as a marker that can be monitored over time to assess β-oxidation and joint health, while also potentially predicting the onset and progression of OA. Furthermore, cervonyl carnitine warrants further investigation as a potential biomarker and druggable target for the purpose of slowing, halting, or reversing OA. 

### 4.3. Vitamin Metabolism

Vitamin E metabolism was notably upregulated in OA cartilage compared to healthy cartilage. Vitamin E has antioxidant properties, which could prove beneficial in counteracting the heightened oxidative stress experienced by the joints during OA [42]. Additionally, vitamin A was dysregulated across OA endotypes. The relationship between OA and vitamin A, including the vitamin A derivative all-trans retinoic acid, has garnered attention due to the key role of this molecule in skeletal development and cartilage maintenance [43,44]. Specifically, all-trans retinoic acid can regulate type X collagen and matrix metalloproteinase-13, driving a hypertrophic phenotype [44,45]. Moreover, elevated vitamin A metabolite levels have been detected in SF, serum, and cartilage from OA individuals, suggesting vitamin A potential role in OA within cartilage [43].

In contrast, vitamin K metabolism was downregulated in OA cartilage compared to healthy cartilage. These findings align with prior research that explored the relationship between OA and vitamin K. Vitamin K is important for its role as a cofactor for the carboxylation of vitamin K-dependent proteins, including matrix Gla proteins, osteocalcin, and Gas-6 [46]. These proteins are present in the joints and play a key role in the maintenance of cartilage and bone. Their absence or deficiency can lead to an increased incidence and progression of knee OA [46,47,48]. Specifically, alterations in vitamin K levels parallel the abnormalities observed in OA disease progression, encompassing aspects such as hypertrophic and apoptotic chondrocytes, cartilage mineralization, and endochondral ossification [49,50]. 

### 4.4. Amino Acid Metabolism

Amino acid metabolism was significantly downregulated in OA cartilage compared to healthy cartilage. While histidine metabolism was not differently regulated across endotypes, its pronounced downregulation in OA cartilage compared to healthy cartilage aligns with findings of a previous study that identified declining trends in serum histidine levels as OA advances [51]. Additionally, the ratio of branched-chain amino acids to histidine has emerged as a potential indicator of disease progression [52]. In contrast, various amino acids including tryptophan, methionine, cysteine, aspartate, and asparagine were upregulated in healthy cartilage compared to OA cartilage, as confirmed by pairwise and endotype comparisons.

This pattern mirrors similar observations made in our prior work comparing SF metabolism in healthy and early- and late-stage OA patients, indicating that these amino acid pathways were upregulated in healthy SF [16]. Focusing solely on OA cartilage, these same amino acid pathways displayed different regulation patterns across the identified OA endotypes. This aligns with previous literature, indicating that the levels of these amino acids tend to decrease as OA progresses, being highest in healthy cartilage, moderately high in early-stage OA, and diminishing in end-stage OA [53,54]. Furthermore, specific amino acids like glycine and alanine, both of which are abundant in collagen, have been putatively identified as potential markers to distinguish osteoarthritic cartilage from healthy cartilage [7]. This observed dysregulation of amino acids may indicate their potential role in responding to the disease and could reflect the degree of joint damage. Nevertheless, further research is required to underpin the relationship between amino acid metabolism and OA. 

### 4.5. Limitations

This study included healthy cartilage samples to examine disease-associated metabolic changes; however, it is not without limitations. Firstly, the sample size of this study was not uniform, as 11 healthy cartilage and 35 OA cartilage samples were obtained. Secondly, relevant clinical covariates (e.g., age, BMI, sex, prior medical history) and the time of death (to calculate the time between death and sample extraction) were not available for the obtained healthy cartilage samples. Furthermore, patient sex and age, with the exception of three patients, were provided for OA donors, yet the BMI was not provided. Considering the partial information provided for both healthy and OA cartilage samples, age-, BMI-, and sex-matching analyses were not performed, nor can this information be used to shed light on driving factors that differentiate OA endotypes. 

## 5. Conclusions

The results of this study provide clear evidence of OA-induced metabolic perturbations in human articular cartilage. Considering the heterogenous nature of OA, the detection of metabolic differences between healthy and OA individuals and within OA individuals alone can be further extended to pinpoint the diverse landscape of OA. With this approach, we uncovered specific metabolomic patterns and identified metabolites that may serve as valuable indicators of disease status or therapeutic targets. The expansion of this study will delineate joint-level metabolic activity in cartilage and how that is reflected by or associated with the metabolism of other musculoskeletal tissues and fluids. 

## Figures and Tables

**Table 1:** Metabolic pathways associated with healthy and diseased cartilage identified by volcano plot analyses. All reported pathways have an FDR-corrected significance level < 0.05.

Group	Regulation	Pathway
Osteoarthritis	FC > 2, *p* < 0.05	Carnitine shuttle
Osteoarthritis	FC > 2, *p* < 0.05	De novo fatty acid biosynthesis
Osteoarthritis	FC > 2, *p* < 0.05	Fatty acid activation
Osteoarthritis	FC > 2, *p* < 0.05	Fatty acid metabolism
Osteoarthritis	FC > 2, *p* < 0.05	Fatty acid oxidation
Osteoarthritis	FC > 2, *p* < 0.05	Fatty acid oxidation, peroxisome
Osteoarthritis	FC > 2, *p* < 0.05	Glycosphingolipid biosynthesis—ganglioseries
Osteoarthritis	FC > 2, *p* < 0.05	Glycosphingolipid biosynthesis—globoseries
Osteoarthritis	FC > 2, *p* < 0.05	Leukotriene metabolism
Osteoarthritis	FC > 2, *p* < 0.05	N-glycan degradation
Osteoarthritis	FC > 2, *p* < 0.05	Omega-3 fatty acid metabolism
Osteoarthritis	FC > 2, *p* < 0.05	Omega-6 fatty acid metabolism
Osteoarthritis	FC > 2, *p* < 0.05	Phosphatidylinositol phosphate metabolism
Osteoarthritis	FC > 2, *p* < 0.05	Phytanic acid peroxisomal oxidation
Osteoarthritis	FC > 2, *p* < 0.05	Polyunsaturated fatty acid biosynthesis
Osteoarthritis	FC > 2, *p* < 0.05	R group synthesis
Osteoarthritis	FC > 2, *p* < 0.05	Saturated fatty acid beta-oxidation
Healthy	FC < −2, *p* < 0.05	Aspartate and asparagine metabolism
Healthy	FC < −2, *p* < 0.05	Glycerophospholipid metabolism
Healthy	FC < −2, *p* < 0.05	Glycine, serine, alanine and threonine metabolism
Healthy	FC < −2, *p* < 0.05	Histidine metabolism
Healthy	FC < −2, *p* < 0.05	Methionine and cysteine metabolism
Healthy	FC < −2, *p* < 0.05	Purine metabolism
Healthy	FC < −2, *p* < 0.05	Squalene and cholesterol biosynthesis
Healthy	FC < −2, *p* < 0.05	Tryptophan metabolism
Healthy	FC < −2, *p* < 0.05	Urea cycle/amino group metabolism
Healthy	FC < −2, *p* < 0.05	Vitamin E metabolism
Healthy	FC < −2, *p* < 0.05	Vitamin K metabolism

**Table 2:** Metabolic pathways associated with osteoarthritis endotypes classified by median intensity heatmap analysis. No significant pathways were detected in cluster 6. All pathways reported has an FDR-corrected significance level < 0.05. Clusters defined in Figure 2E.

Cluster	Pathway
1	Fatty acid activation
1	Saturated fatty acids beta-oxidation
1	De novo fatty acid biosynthesis
1	Fatty acid metabolism
1	Omega-6 fatty acid metabolism
1	Carnitine shuttle
1	R group synthesis
1	Fatty acid oxidation
1	Fatty acid oxidation, peroxisome
1	Leukotriene metabolism
2	Fatty acid oxidation
2	Polyunsaturated fatty acid biosynthesis
2	De novo fatty acid biosynthesis
2	Phytanic acid peroxisomal oxidation
2	R group synthesis
2	Selenoamino acid metabolism
3	Phytanic acid peroxisomal oxidation
3	Omega-6 fatty acid metabolism
4	Glycosphingolipid biosynthesis—globoseries
4	Lysine metabolism
4	Tyrosine metabolism
4	Polyunsaturated fatty acid biosynthesis
4	Glycosphingolipid biosynthesis—ganglioseries
4	Keratan sulfate degradation
4	N-glycan degradation
4	Linoleate metabolism
4	Vitamin A (retinol) metabolism
4	Butanoate metabolism
4	Trihydroxycoprostanoyl-CoA beta-oxidation
4	Glycerophospholipid metabolism
4	Omega-3 fatty acid metabolism
4	Starch and sucrose metabolism
5	Purine metabolism
5	Leukotriene metabolism
5	Urea cycle/amino group metabolism
5	Methionine and cysteine metabolism
5	Tryptophan metabolism
5	Aminosugar metabolism
5	Aspartate and asparagine metabolism

## Data Availability

The original contributions presented in the study are included in the article/Supplementary Material. Further inquiries can be directed to the corresponding author/s.

## Supplementary Materials

The following supporting information can be downloaded at: https://www.mdpi.com/article/10.3390/metabo14040183/s1, Figure S1: Identified metabolites differ in abundance between healthy and osteoarthritic cartilage. Figure S2: Ensemble clustering of osteoarthritis participants revealed distinct metabolic endotypes. Figure S3: Identified metabolites differ in abundance across osteoarthritis endotypes. Table S1: Participant information including donor number, assigned anonymous identifier, age, sex, and osteoarthritis endotype group. Table S2: All metabolic pathways determined from MetaboAnalyst when comparing healthy and diseased cartilage using volcano plot analyses. Populations of metabolite features are defined on Figure 1C. Table S3: Putatively identified metabolites that differ in abundance between healthy and diseased cartilage distinguished by volcano plot analysis. Identifications were made by performing liquid chromatography tandem mass spectrometry (LC-MS/MS). For all identified, information includes observed and theoretical mass-to-charge ratios, parts per million (ppm) error, accepted compound ID and description, adduct, chemical formula, total score out of 100, and fragmentation score. Identifications with error greater than 20 ppm, total score < 60, and a fragmentation score < 12 were excluded. Table S4: Putatively identified metabolites that differ in abundance between healthy and diseased cartilage distinguished by t-test analysis. Identifications were made by performing liquid chromatography tandem mass spectrometry (LC-MS/MS). For all identified, information includes observed and theoretical mass-to-charge ratios, parts per million (ppm) error, accepted compound ID and description, adduct, chemical formula, total score out of 100, and fragmentation score. Identifications with error greater than 20 ppm, total score < 60, and a fragmentation score < 12 were excluded. Table S5: Putatively identified metabolites that differ in abundance between osteoarthritis endotype groups distinguished by ANOVA analysis. Identifications were made by performing liquid chromatography tandem mass spectrometry (LC-MS/MS). For all identified, information includes observed and theoretical mass-to-charge ratios, parts per million (ppm) error, accepted compound ID and description, adduct, chemical formula, total score out of 100, and fragmentation score. Identifications with error greater than 20 ppm, total score < 60, and a fragmentation score < 12 were excluded. All metabolite features with an FDR-corrected *p*-value > 0.05 distinguished by ANOVA when comparing all four osteoarthritis groups. Table S6: All metabolic pathways determined from MetaboAnalyst when comparing osteoarthritis endotypes using median metabolite intensity heatmap analysis. Clusters defined on Figure 2E. Table S7: Raw data from XCMS.
