# Supplementary material for: Metabolomic Profiles and Pathways in Osteoarthritic Human Cartilage: A Comparative Analysis with Healthy Cartilage
Source: Metabolites. 2024 Mar 25;14(4):183. doi: 10.3390/metabo14040183 (PMC11051929; doi:10.3390/metabo14040183)
Supplement: Supplementary file 1 [file metabolites-14-00183-s001.zip › HealthyvsDiseased_MDPI_Tables_vRev.pdf]

Highest In	m/z	Progenesis m/z	Mass Error (ppm)
Healthy	119.060069	119.0595711	-6.830267195
	119.060069	119.0595711	-6.806566327
Healthy	130.024509	130.0235934	-8.711911535
Healthy	164.014983	164.0141201	0.458091397
Healthy	170.030653	170.0298536	9.432038673
Healthy	210.063548	210.0625986	-6.328971744
Healthy	252.084856	252.0840623	-6.623526452
Healthy	263.103186	263.1017605	-3.330165563
Healthy	268.082227	268.0807126	0.872769285
Healthy	273.145904	273.146731	2.461545549
	273.145904	273.146731	2.461545549
	273.145904	273.146731	2.461545549
	273.145904	273.146731	2.475793523
	273.145904	273.146731	2.461545549
	273.145904	273.146731	2.461545549
	273.145904	273.146731	2.475793523
	273.145904	273.146731	2.475793523
	273.145904	273.146731	2.461545549
	273.145904	273.146731	2.461545549
Healthy	280.117843	280.1174785	-1.686104569
Healthy	299.101586	299.1003539	0.511285275
Healthy	305.13762	305.1368575	3.235847782
	305.13762	305.1378272	6.672911318
Healthy	313.069919	313.0673012	-3.311231931
	313.069919	313.0673012	-3.301463384
Healthy	317.188902	317.1885518	8.208634718
	317.188902	317.1885518	8.208634718
Healthy	321.113237	321.1121698	8.184987438
	321.113237	321.1121698	0.10754214
	321.113237	321.1121698	8.184987438

Healthy	327.23224	327.2321191	8.769987921
Healthy	341.248911	341.2483666	2.516244613
	341.248911	341.2483666	2.516244613
Healthy	343.114548	343.1143175	-2.784965477
	343.114548	343.1143175	-2.784965477
Healthy	347.165846	347.166375	3.860111469
Healthy	348.193673	348.1932211	-7.443709246
	348.193673	348.1932211	-7.443709246
	348.193673	348.1932211	-7.443709246
Healthy	349.16684	349.1663159	5.030654473
Healthy	355.265651	355.2655095	6.614082099
Healthy	357.131336	357.1308988	-6.64382805
	357.131336	357.1308988	-6.64382805
	357.131336	357.1308988	-6.64382805
	357.131336	357.1308988	-6.64382805
	357.131336	357.1308988	-6.64382805
	357.131336	357.1308988	-6.64382805
	357.131336	357.1308988	-6.64382805
	357.131336	357.1308988	-6.64382805
	357.131336	357.1308988	9.55702194
	357.131336	357.1308988	-7.304142567
Healthy	363.197939	363.1971633	1.534760866
Healthy	365.140092	365.1377348	5.232545476
Healthy	371.188703	371.1886024	-0.186641035
	371.188703	371.1886024	-0.186641035
Healthy	379.137695	379.1385626	6.24507266
Healthy	379.231064	379.2311824	8.629853743
Healthy	385.278105	385.2773501	9.443153923
	385.278105	385.2773501	9.443153923
Healthy	387.234302	387.2344285	3.738887609
	387.234302	387.2344285	-8.507733111

Healthy	389.177112	389.1755807	5.221511408
Healthy	399.295298	399.2951803	-3.522801376
Healthy	413.254223	413.2537177	0.842359691
Healthy	425.203748	425.2033123	-8.925369221
Healthy	430.232803	430.2330325	4.397132381
Healthy	437.183871	437.1834451	-7.910609949
	437.183871	437.1834451	-0.269293361
Healthy	439.19096	439.1901962	-8.786680875
	439.19096	439.1901962	3.05762053
	459.3214	459.3208708	-5.684942744
	459.3214	459.3208708	-5.684942744
Healthy	479.195704	479.1995112	-9.912995468
	479.195704	479.1995112	0.010022384
Healthy	482.258846	482.2553504	-3.605288388
Healthy	489.336073	489.3349943	-2.71827169
Healthy	501.300337	501.3034363	-4.743447495
	501.367472	501.3683499	-4.079609747
	501.367472	501.3673663	2.273297588
Healthy	504.266603	504.2659741	4.100328154
Healthy	507.290213	507.2898201	2.173155019
	507.324196	507.3244679	2.305344108
Healthy	510.341826	510.341302	-2.435369387
Healthy	531.31211	531.3122366	-7.802110291
Healthy	540.289482	540.291902	8.071034309
Healthy	545.396299	545.396629	0.215732749
Healthy	565.371599	565.3708039	6.817390546
Healthy	569.247956	569.252058	-8.767634864

	569.247956	569.252058	1.983940819
Healthy	573.403851	573.4045509	-3.905738475
	573.403851	573.4045509	-3.880941648
Healthy	579.278966	579.2782817	-1.100059738
Healthy	579.388397	579.3876181	-2.689037335
	579.388397	579.3876181	-2.689037335
Healthy	585.377152	585.3748861	-2.29144377
Healthy	586.309961	586.3105491	-2.939211218
Healthy	589.425081	589.4257097	0.973775854
Healthy	591.410497	591.4121109	-9.059758857
Healthy	593.368842	593.3686187	0.352843685
	593.410783	593.4057744	1.652903305
Healthy	605.427726	605.428854	-6.922000935
	605.427726	605.428854	-6.922000935
	605.427726	605.428854	-6.922000935
	605.427726	605.4275945	6.949896727
	605.427726	605.428854	4.860161835
Healthy	611.314133	611.3184349	-0.597294864
Healthy	617.334264	617.3318838	3.819567529
Healthy	621.430302	621.4256961	-3.543081467
Healthy	625.327497	625.327167	-0.074668329
Healthy	635.41557	635.414837	-0.824194362
	635.440265	635.4424264	0.914133286
Healthy	675.43831	675.4377729	-9.933806486
Healthy	694.471255	694.4658626	-2.692605862
Healthy	707.503465	707.5073679	-6.123721697
Healthy	709.486551	709.4879768	2.70599108

	709.486551	709.4879768	2.70599108
Healthy	721.408248	721.4068243	-9.340737448
	721.408248	721.4068243	-9.340737448
	721.408248	721.4068243	-9.340737448
Healthy	723.50207	723.5063555	6.547128916
	723.50207	723.5063555	6.547128916
Healthy	737.422857	737.4231864	-0.061114298
Healthy	749.427998	749.4289584	7.65210413
	749.54639	749.5473193	-0.849696537
Healthy	765.440198	765.4410386	-1.216569041
	765.440198	765.4410386	1.987608054
Healthy	771.508782	771.5100644	-6.156492176
Healthy	809.471669	809.472784	5.690654565
	809.471669	809.472784	8.908542218
	809.471669	809.472784	8.908542217
Healthy	869.517032	869.5188128	-7.927762579
Healthy	958.578934	958.5880737	-0.583806249
Osteoarthritis	178.033845	178.0327355	3.525957082
Osteoarthritis	220.043408	220.0441048	-8.448698136
Osteoarthritis	369.122538	369.1291446	-0.264634679
Osteoarthritis	405.197126	405.1947228	-8.059643142
Osteoarthritis	464.305935	464.3060359	-3.968404957
Osteoarthritis	466.321431	466.3122466	-8.74246491
	466.321431	466.3122466	-8.74246491
	466.321431	466.3122466	-8.74246491
Osteoarthritis	487.29224	487.2844717	5.571900724
Osteoarthritis	494.312139	494.3205418	-7.505881935
Osteoarthritis	552.07514	552.0735244	-3.66525424

Osteoarthritis	790.49629	790.49399	-0.903641817
Osteoarthritis	829.469017	829.4554053	-3.147129513
Osteoarthritis	1071.67518	1071.668515	6.597887384

Compound ID	Description
CSID5593	Benzimidazole
HMDB40040	5H-Cyclopentapyrazine
HMDB41861, HMDB0041861_c_ms_102279	cyanuric acid
HMDB37168	2-Propanoylthiazole
CSID6025	Methylnitronitrosoguanidine
HMDB15496,CSID3570	Amrinone
CSID20129465	N-Hydroxy-meIQX
HMDB31360	L-cis-Cyclo(aspartylphenylalanyl)
CSID4450275	(4Z)-4-(2-Amino-5-oxo-1,5-dihydro-4H-imidazo
HMDB35798, CSID143579	Piperdial
HMDB31901	Blennin A
HMDB37529	Lactaronecatorin A
CSID65413	Warburganal
HMDB39644	Heliannuol A
HMDB35137	Heliannuol D
CSID30790779	(1R,3aR,5aS,6R)-6-Hydroxy-1,7,7-trimethyl-1,2,[illegible]
CSID30790781	(1S,2R,3aR,5aS)-2-Hydroxy-1,7,7-trimethyl-1,2,[illegible]
HMDB30917	1-Hydroxyacorenone
HMDB35148	(6beta,8alpha)-6-Hydroxy-7(11)-eremophilen-1
HMDB32807	Niazirin
CSID38263	Oxagrelate
HMDB33375	Octyl gallate
HMDB38989	Oxyhumulinic acid
CSID389080	(+)-leucopelargonidin
HMDB32322	cis-3 and trans-2-Hexenyl propionate
CSID26433	amquinate
CSID54820	5-{3-[(2S)-Bicyclo[2.2.1]hept-2-yloxy]-4-methox
HMDB29543	(+)-Ligballinol
HMDB41463	2,3-Dihydro-2,3-dihydroxy-4-(4-methoxyphenyl
HMDB04808	7C-aglycone

HMDB01043, CSID392692	Arachidonic acid
CSID6357	dimethisterone
CSID140149	17-ethynylandrost-2-ene-17-ol-17-acetate
HMDB15159	Mycophenolic acid
HMDB37321	Oleacein
CSID8134	Piperonyl sulfoxide
HMDB38837	(R)-Mahanine
HMDB38827	Murrayazolinol
HMDB38828	Pyrafoline D
CSID4444835	Tagitinin F
CSID30791731	Bufa-20,22-dienolide
HMDB41290	1,8-Dihydroxy-3,5-dimethoxy-2-prenylxanthone
HMDB29459	Xanthoxylol
HMDB30682	Cubebin
HMDB38774	5-Deoxykievitol
HMDB33027	Licoagrodione
HMDB35525	Licofuranone
HMDB34213	Kievitone
HMDB41123	Kinobeon A
HMDB12996	Lipoyllysine
HMDB41826	amifloxacin
CSID210391	17,21-Epoxy-9-fluoro-11beta-hydroxyprogester
HMDB31535	Hydroxygaleon
HMDB02759	Androsterone sulfate
HMDB13232	Etiocholanolone sulfate
HMDB31695	xi-3-Hydroxy-5-phenylpentanoic acid O-beta-D-
CSID10128451	21-fluoro-11?,17-dihydroxy-6?-methylprogeste
HMDB34952	Persicaxanthin
HMDB36425	Persicachrome
CSID4940685	dalvastatin
CSID20016206	oleandolide

CSID29271896	Liafensine
CSID2981	Dimorpholamine
CSID390585	(14R)-3beta,5,6beta,10,16-pentahydroxygrayar
HMDB14682	Benazepril
HMDB13024	Neurotensin 11-13
HMDB15013	Flupenthixol
CSID4593753	naltrindole
HMDB36340	Ethyl 7-epi-12-hydroxyjasmonate glucoside
CSID9399462	Radezolid
HMDB35983	Ganoderal A
HMDB30395	Manglupenone
CSID23332653	kurarinol
CSID64481	Clobetasone butyrate
HMDB05084	N-Acetyl-leukotriene E4
HMDB36884	7',8'-Dihydro-8'-hydroxycitraniaxanthin
HMDB14554	Mupirocin
HMDB34578	Deterrol stearate
HMDB30272	Carpaine
CSID447456	ANTIBIOTIC JI-20A
HMDB15593	Ixabepilone
HMDB39015	Tangeraxanthin
CSID4445266	narbomycin
HMDB40844	(S)-Nerolidol 3-O-[a-L-rhamnopyranosyl-(1->2)-
CSID2339128	Apramycin
HMDB41205	Panaxydol linoleate
CSID391760	(1S,2R,3S,4S,6R,7R,8R,14S)-3-Hydroxy-2,4,7,14-
HMDB36317	Canavalioside

HMDB34352	Morellinol
CSID4444648	Echinenone
HMDB02890	3-cis-Hydroxy-b,e-Caroten-3'-one
CSID20131003	Omadacycline
CSID30791712	Timosaponin A-I
CSID389388	(25S)-5beta-spirostan-3beta-yl beta-D-glucoside
HMDB10330	Cholesterol glucuronide
CSID21106220	Tanespimycin
HMDB40807	Caffeoylcycloartenol
CSID4444655	Lutein
HMDB34391	Agavoside A
HMDB39054	Tuberoside
CSID4518328	Hydroxyspirilloxanthin
CSID4518272	Spheroidenone
CSID30791959	Thiothece-474
CSID570993	Polidocanol
CSID4445232	3,6-Anhydro-2,4,5-tris-O-(2-hydroxyethyl)-1-O-
CSID391535	BQ-123
CSID10473090	13-deoxytedanolide
HMDB30100	(8'R)-Neochrome
HMDB34104	Neferine
HMDB35917	Momordicoside L
CSID148923	Eldacimibe
HMDB38016	Ginsenoside Rh5
HMDB30492	Spirolide B
CSID8093615	trodusquemine
MGDG(26:1/3:0)	MGDG(26:1/3:0)

MGDG(3:0/26:1)	MGDG(3:0/26:1)
HMDB35944	Cyclopassifloside I
HMDB35946	Cyclopassifloside IV
HMDB36365	Cyclopassifloside X
MGDG(26:1/4:0)	MGDG(26:1/4:0)
MGDG(4:0/26:1)	MGDG(4:0/26:1)
CSID30792054	Myxochromide S2
CSID30792053	Myxochromide S3
CSID4446659	Ubiquinone-10
HMDB15468	Digitoxin
HMDB38345	Alliosterol 1-rhamnoside 16-galactoside
CSID4445464	1-PALMITOYL-2-OLEOYL-SN-GLYCERO-3-(PHOSP
HMDB41018	Calenduloside G methyl ester
HMDB40781	Vinaginsenoside R11
HMDB40418	Majonoside R2
HMDB41351	Koryoginsenoside R1
HMDB15529	Everolimus
HMDB38591	Brassicanal A
CSID389199	Guanidinoethyl methyl phosphate
HMDB01204	trans-3-Hydroxycotinine glucuronide
CSID108701	Tetraxetan
CSID388706	Gentamycin C2
HMDB00138	Glycocholic acid
HMDB00331	3a,7b,12a-Trihydroxyoxocholanyl-Glycine
HMDB32596	Sodium glycocholate
CSID140269	Pristimerin
HMDB06510	Cervonyl carnitine
CSID30791094	dTDP-3-amino-2,3,6-trideoxy-D-threo-hexopyra

CSID64402	ERYTHROMYCIN PROPIONATE
HMDB35449	1beta,2alpha,3beta,19alpha-Tetrahydroxy-12-u
CSID28285403	(3S)-3-{(2S,3R,5S)-5-[(2R)-3-{(2S,4aS,5aR,6R,6aS

Accepted?	Adducts	Formula	Score (out of 100)	Score (out of 60)	Fragmentation Score
	M+H	C7H6N2	72.66666667	43.6	27.8
	M+H	C7H6N2	73.66666667	44.2	30.7
	M+H	C3H3N3O3	71.5	42.9	28.9
*	M+Na	C6H7NOS	70.33333333	42.2	23.4
*	M+Na	C2H5N5O3	67.16666667	40.3	15.2
	M+Na	C10H9N3O	69.16666667	41.5	19
*	M+Na	C11H11N5O	71.5	42.9	35.2
*	M+H, M+Na	C13H14N2O4	70.5	42.3	19.9
*	M+Na	C11H11N5O2	67.16666667	40.3	15.5
	M+Na	C15H22O3	71.66666667	43	22.8
	M+Na	C15H22O3	71.66666667	43	22.8
	M+Na	C15H22O3	71.83333333	43.1	23.4
	M+Na	C15H22O3	71.83333333	43.1	23.4
	M+Na	C15H22O3	72	43.2	23.8
	M+Na	C15H22O3	72	43.2	23.8
3,3a,5a,6,7,8-	M+Na	C15H22O3	72	43.2	23.8
3,3a,5a,6,7,8-	M+Na	C15H22O3	72	43.2	24.1
	M+Na	C15H22O3	72.33333333	43.4	25.1
2,8-olide	M+Na	C15H22O3	72.5	43.5	25.3
*	M+H	C14H17NO5	73	43.8	23.6
*	M+Na	C14H16N2O4	70.33333333	42.2	17.5
*	M+Na	C15H22O5	67	40.2	15
*	M+Na	C15H22O5	67.33333333	40.4	25.6
	M+Na	C15H14O6	69.5	41.7	15.9
	M+Na	C15H14O6	69.5	41.7	15.9
	M+H	C18H24N2O3	67.5	40.5	16.6
yphenyl}tetra	M+H	C18H24N2O3	67.5	40.5	16.6
	M+Na	C18H18O4	68.33333333	41	23.3
)-1H-phenale	M+H	C20H16O4	68.66666667	41.2	17
	M+Na	C18H18O4	69.16666667	41.5	25.4

	M+Na	C20H32O2	66.83333333	40.1	13
	M+H	C23H32O2	70	42	16.5
	M+H	C23H32O2	70.16666667	42.1	16.9
	M+Na	C17H20O6	68.16666667	40.9	14.7
	M+Na	C17H20O6	69.5	41.7	18.8
*	M+H, M+Na	C18H28O3S	69.33333333	41.6	16.4
	M+H	C23H25NO2	67.33333333	40.4	13.2
	M+H	C23H25NO2	67.5	40.5	13.8
	M+H	C23H25NO2	67.5	40.5	13.8
*	M+H	C19H24O6	67.16666667	40.3	27.4
*	M+H	C24H34O2	70.66666667	42.4	24.5
e	M+H	C20H20O6	67.5	40.5	16.5
	M+H	C20H20O6	67.83333333	40.7	17.5
	M+H	C20H20O6	68	40.8	18.1
	M+H	C20H20O6	68.16666667	40.9	18.6
	M+H	C20H20O6	68.33333333	41	19
	M+H	C20H20O6	68.5	41.1	19.2
	M+H	C20H20O6	68.83333333	41.3	20.6
	M+H	C20H20O6	69.33333333	41.6	21.9
	M+Na	C14H26N2O3	73.16666667	43.9	33.8
	M+Na	C16H19FN4C	75.33333333	45.2	40.8
*	M+H	C21H27FO4	71.33333333	42.8	26.3
*	M+Na	C20H22O5	67	40.2	12.8
	M+H, M+Na	C19H30O5S	72	43.2	19.6
	M+H, M+Na	C19H30O5S	72	43.2	19.6
Glucopyranos	M+Na	C17H24O8	67.83333333	40.7	17.7
*	M+H	C22H31FO4	68.5	41.1	24.7
	M+H	C25H36O3	67.66666667	40.6	17.5
	M+H	C25H36O3	67.66666667	40.6	17.5
	M+H	C24H31FO3	69	41.4	18.7
	M+H	C20H34O7	70.83333333	42.5	25.8

*	M+Na	C24H22N4	68	40.8	25.2
*	M+H	C20H38N4O4	70	42	17.8
*	M+H	C22H36O7	76.33333333	45.8	40.4
	M+H	C24H28N2O5	73.33333333	44	32.8
*	M+Na	C21H33N3O5	67.66666667	40.6	22.1
	M+H	C23H27F3N2	71	42.6	29.9
	M+Na	C26H26N2O3	75.66666667	45.4	32.2
	M+Na	C20H32O9	67.33333333	40.4	20.2
	M+H	C22H23FN6O	72	43.2	30.2
	M+Na	C30H44O2	72.33333333	43.4	28.4
	M+Na	C30H44O2	74.16666667	44.5	34.2
	M+Na	C26H32O7	72	43.2	39.2
	M+H	C26H32ClFO5	78.16666667	46.9	51.8
	M+H	C25H39NO6S	77.83333333	46.7	49.9
*	M+H	C33H44O3	68.33333333	41	19.2
	M+H	C26H44O9	71.83333333	43.1	31.5
	M+Na	C33H50O2	67.66666667	40.6	16.8
	M+Na	C28H50N2O4	69	41.4	19.9
*	M+Na	C19H39N5O9	68	40.8	18.2
	M+H, M+Na	C27H42N2O5	71	42.6	21.4
*	M+Na	C34H44O2	69.16666667	41.5	20.7
*	M+H	C28H47NO7	67	40.2	25.3
*	M+H	C27H46O10	67	40.2	12.3
*	M+H	C21H41N5O1	68.66666667	41.2	33.2
*	M+Na	C35H54O3	69.33333333	41.6	20
*	M+H	C31H52N2O5	70.5	42.3	29.2
	M+Na	C26H42O12	67.33333333	40.4	22.5

	M+Na	C33H38O7	71.33333333	42.8	24
	M+Na	C40H54O	71	42.6	25
	M+Na	C40H54O	71.66666667	43	26.8
*	M+H, M+Na	C29H40N4O7	68.66666667	41.2	17.9
	M+H	C33H54O8	69	41.4	20.4
e	M+H	C33H54O8	69	41.4	20.4
*	M+Na	C33H54O7	67.66666667	40.6	12.4
*	M+H	C31H43N3O8	69.16666667	41.5	17
*	M+H, M+Na	C39H56O4	77	46.2	41.8
	M+Na	C40H56O2	79	47.4	58.4
	M+H	C33H52O9	71.66666667	43	32.4
*	M+H	C34H56O8	70.5	42.3	22.3
	M+Na	C41H58O2	70.16666667	42.1	26.8
	M+Na	C41H58O2	71	42.6	29.4
	M+Na	C41H58O2	72.33333333	43.4	33.3
	M+Na	C30H62O10	74	44.4	37.4
{2-[(9E)-9-oct	M+H	C32H60O10	75.33333333	45.2	37
*	M+H, M+Na	C31H42N6O7	70.16666667	42.1	24.1
*	M+Na	C32H50O10	67	40.2	29.5
*	M+Na	C41H58O3	74.5	44.7	38.4
	M+H, M+Na	C38H44N2O6	71	42.6	18.3
	M+H, M+Na	C36H58O9	68.16666667	40.9	15.8
*	M+H	C39H58N2O5	76.16666667	45.7	41.9
*	M+Na	C37H64O9	68.16666667	40.9	27
*	M+H	C42H63NO7	69.16666667	41.5	30.4
*	M+Na	C37H72N4O5	67.83333333	40.7	29.6
	M+Na	C38H70O10	68.66666667	41.2	26.4

	M+Na	C38H70O10	68.66666667	41.2	26.4
	M+Na	C37H62O12	72	43.2	37.7
	M+Na	C37H62O12	72	43.2	37.7
	M+Na	C37H62O12	73.83333333	44.3	43.2
	M+Na	C39H72O10	69.16666667	41.5	26.2
	M+Na	C39H72O10	69.16666667	41.5	26.2
*	M+H	C39H56N6O8	69.66666667	41.8	12.7
*	M+H	C40H56N6O8	68.5	41.1	23.9
*	M+Na	C49H74O4	68.33333333	41	17.1
	M+H	C41H64O13	68.83333333	41.3	15.4
	M+Na	C39H66O13	74.33333333	44.6	33.3
*	M+Na	C40H77O10P	70.66666667	42.4	20.6
	M+H	C43H68O14	68.33333333	41	15.4
	M+Na	C41H70O14	72.5	43.5	30.4
	M+Na	C41H70O14	73.16666667	43.9	32
*	M+H	C46H76O15	70	42	30.9
	M+H	C53H83NO14	69.16666667	41.5	27.8
	M+H	C9H7NOS	63.66666667	38.2	15.6
	M+Na	C4H12N3O4P	70.33333333	42.2	26.5
*	M+H	C16H20N2O8	67.16666667	40.3	14.5
	M+H	C16H28N4O8	66.66666667	40	18.6
	M+H, M+Na	C20H41N5O7	66.83333333	40.1	14.3
	M+H	C26H43NO6	65.16666667	39.1	15.5
	M+H	C26H43NO6	65.16666667	39.1	15.5
	M+H	C26H43NO6	65.16666667	39.1	15.5
	M+Na	C30H40O4	66.5	39.9	14
	M+Na	C29H45NO4	66.16666667	39.7	14.7
anos-4-ulose	M+Na	C16H25N3O1	64.33333333	38.6	16.9

*	M+H	C40H71NO14	69.5	41.7	47.6
ırsen-28-oic a	M+H	C42H68O16	63.16666667	37.9	14.2
;,7aR,8R,9Z,11	M+H	C60H94O16	62.83333333	37.7	14.6

otope Similari	cal Isotope Dis	Link	Description.1	eutral mass (D	Charge	ention time (m
98.0311493	100 - 8.37 - 0	http://nonlin	Benzimidazole		1	0.73403333
98.0311493	100 - 8.37 - 0	http://nonlin	5H-Cyclopentapyrazine		1	0.73403333
95.0686083	100 - 4.49 - 0	http://nonlin	cyanuric acid		1	0.73403333
88.4189908	100 - 7.77 - 4	http://nonlin	2-Propanoylthiazole		1	0.73403333
96.4061201	100 - 4.16 - 0	http://nonlin	Methylnitronitrosoguanidi		1	4.13171667
95.5626578	100 - 12.1 - 0	http://nonlin	Amrinone		1	0.73403333
86.9634587	100 - 13.9 - 1	http://nonlin	N-Hydroxy-meIQX		1	4.166
95.4819316	100 - 15.1 - 1	http://nonlin	L-cis-Cyclo(aspartylphenyla		1	4.166
86.7756443	100 - 13.9 - 1	http://nonlin	(4Z)-4-(2-Amino-5-oxo-1,5-		1	4.166
94.9594626	100 - 16.6 - 1	http://nonlin	Piperdial		1	4.166
94.9594626	100 - 16.6 - 1	http://nonlin	Blennin A		1	4.166
94.9594626	100 - 16.6 - 1	http://nonlin	Lactaronecatorin A		1	4.166
94.9594626	100 - 16.6 - 1	http://nonlin	Warburganal		1	4.166
94.9594626	100 - 16.6 - 1	http://nonlin	Heliannuol A		1	4.166
94.9594626	100 - 16.6 - 1	http://nonlin	Heliannuol D		1	4.166
94.9594626	100 - 16.6 - 1	http://nonlin	(1R,3aR,5aS,6R)-6-Hydroxy		1	4.166
94.9594626	100 - 16.6 - 1	http://nonlin	(1S,2R,3aR,5aS)-2-Hydroxy		1	4.166
94.9594626	100 - 16.6 - 1	http://nonlin	1-Hydroxyacorenone		1	4.166
94.9594626	100 - 16.6 - 1	http://nonlin	(6beta,8alpha)-6-Hydroxy-		1	0.73403333
97.2776318	100 - 15.9 - 2	http://nonlin	Niazirin		1	0.73403333
93.9140816	100 - 16.2 - 2	http://nonlin	Oxagrelate	463.298759	1	4.27743333
89.8131977	100 - 16.7 - 2	http://nonlin	Octyl gallate	463.298759	1	4.27743333
83.8716609	100 - 16.7 - 2	http://nonlin	Oxyhumulinic acid		1	0.88831667
96.3145769	100 - 16.6 - 2	http://nonlin	(+)-leucopelargonidin		1	0.7426
96.3145769	100 - 16.6 - 2	http://nonlin	cis-3 and trans-2-Hexenyl p		1	4.14885
94.7863584	100 - 20.6 - 2	http://nonlin	amquinate		1	0.67403333
94.7863584	100 - 20.6 - 2	http://nonlin	5-{3-[(2S)-Bicyclo[2.2.1]hep		1	0.71688333
90.952671	100 - 19.8 - 2	http://nonlin	(+)-Ligballinol		1	0.69975
89.0415486	100 - 22 - 3.1	http://nonlin	2,3-Dihydro-2,3-dihydroxy-		1	8.99353333
90.952671	100 - 19.8 - 2	http://nonlin	7C-aglycone		1	4.166

97.2802588	100 - 22.1 - 2	http://nonlin	Arachidonic acid		1	0.73403333
96.3692067	100 - 25.3 - 3	http://nonlin	dimethisterone		1	4.166
96.3692067	100 - 25.3 - 3	http://nonlin	17-ethynylandrost-2-ene-1		1	0.73403333
92.9086549	100 - 18.8 - 2	http://nonlin	Mycophenolic acid		1	4.14028333
92.9086549	100 - 18.8 - 2	http://nonlin	Oleacein		1	4.24313333
96.1314827	100 - 20.7 - 7	http://nonlin	Piperonyl sulfoxide		1	4.24313333
96.9667703	100 - 25.6 - 3	http://nonlin	(R)-Mahanine		1	0.69975
96.9667703	100 - 25.6 - 3	http://nonlin	Murrayazolinol		1	0.77688333
96.9667703	100 - 25.6 - 3	http://nonlin	Pyrafoline D		1	0.77688333
80.1379613	100 - 21.1 - 3	http://nonlin	Tagitinin F		1	4.23456667
95.0276132	100 - 26.4 - 3	http://nonlin	Bufa-20,22-dienolide		1	0.73403333
93.625403	100 - 22.1 - 3	http://nonlin	1,8-Dihydroxy-3,5-dimetho		1	0.73403333
93.625403	100 - 22.1 - 3	http://nonlin	Xanthoxylol		1	0.69116667
93.625403	100 - 22.1 - 3	http://nonlin	Cubebin		1	0.71688333
93.625403	100 - 22.1 - 3	http://nonlin	5-Deoxykievitol		1	0.72545
93.625403	100 - 22.1 - 3	http://nonlin	Licoagrodione		1	0.67403333
93.625403	100 - 22.1 - 3	http://nonlin	Licofuranone		1	0.73403333
93.625403	100 - 22.1 - 3	http://nonlin	Kievitone		1	4.23456667
93.625403	100 - 22.1 - 3	http://nonlin	Kinobeon A		1	4.23456667
96.1566677	100 - 17.9 - 1	http://nonlin	Lipoyllysine		1	4.23456667
93.4384955	100 - 19.1 - 2	http://nonlin	amifloxacin		1	0.73403333
89.5030534	100 - 23.2 - 3	http://nonlin	17,21-Epoxy-9-fluoro-11be		1	4.20885
94.080151	100 - 22.1 - 3	http://nonlin	Hydroxygaleon		1	4.13171667
96.6508519	100 - 21.9 - 7	http://nonlin	Androsterone sulfate		1	0.73403333
96.6508519	100 - 21.9 - 7	http://nonlin	Etiocholanolone sulfate		1	4.20885
92.9518421	100 - 19 - 3.3	http://nonlin	xi-3-Hydroxy-5-phenylpent		1	4.20885
90.349871	100 - 24.3 - 3	http://nonlin	21-fluoro-11?,17-dihydrox		1	4.20885
95.9553369	100 - 27.6 - 4	http://nonlin	Persicaxanthin		1	4.20885
95.9553369	100 - 27.6 - 4	http://nonlin	Persicachrome		1	0.73403333
92.8665893	100 - 26.4 - 3	http://nonlin	dalvastatin		1	4.20885
95.990098	100 - 22.3 - 3	http://nonlin	oleandolide		1	0.73403333

84.6257574	100 - 27.7 - 3	http://nonlin	Liafensine		1	0.73403333
96.4052544	100 - 23.7 - 3	http://nonlin	Dimorpholamine		1	0.73403333
89.8179674	100 - 24.5 - 4	http://nonlin	(14R)-3beta,5,6beta,10,16		1	0.67403333
96.9814156	100 - 27.2 - 4	http://nonlin	Benazepril		1	4.20885
85.9031001	100 - 24.4 - 3	http://nonlin	Neurotensin 11-13		1	0.73403333
92.0574054	100 - 26.8 - 8	http://nonlin	Flupenthixol		1	4.20885
94.9854272	100 - 29.3 - 4	http://nonlin	naltrindole		1	0.73403333
91.8102661	100 - 22.3 - 4	http://nonlin	Ethyl 7-epi-12-hydroxyjasn		1	0.73403333
89.2721821	100 - 26.4 - 3	http://nonlin	Radezolid		1	0.73403333
94.9882038	100 - 33 - 5.6	http://nonlin	Ganoderal A	506.282544	1	0.71688333
94.9882038	100 - 33 - 5.6	http://nonlin	Manglupenone		1	4.20028333
87.8211124	100 - 28.8 - 5	http://nonlin	kurarinol		1	4.24313333
82.7208612	100 - 28.7 - 3	http://nonlin	Clobetasone butyrate		1	4.24313333
87.9123586	100 - 28.9 - 9	http://nonlin	N-Acetyl-leuk	370.181326	1	4.14885
89.2081678	100 - 36.3 - 7	http://nonlin	7',8'-Dihydro-8'-hydroxycit		1	4.14885
89.5925864	100 - 29 - 5.8	http://nonlin	Mupirocin		1	4.406
90.7330712	100 - 36.3 - 6	http://nonlin	Deterrol stearate		1	4.406
89.8592659	100 - 31.7 - 5	http://nonlin	Carpaine		1	4.20885
90.4447706	100 - 23.2 - 4	http://nonlin	ANTIBIOTIC JI-20A		1	4.20885
93.9299414	100 - 31.4 - 1	http://nonlin	Ixabepilone		1	4.166
89.6012923	100 - 37.4 - 7	http://nonlin	Tangeraxanthin		1	4.20885
78.7843555	100 - 31.5 - 6	http://nonlin	narbomycin		1	8.37325
97.3873371	100 - 30.1 - 6	http://nonlin	(S)-Nerolidol 3-O-[a-L-rhan		1	0.73403333
82.0020277	100 - 25.4 - 5	http://nonlin	Apramycin		1	0.73403333
88.2589798	100 - 38.6 - 7	http://nonlin	Panaxydol linoleate		1	0.73403333
90.2612223	100 - 35.8 - 1	http://nonlin	(1S,2R,3S,4S,6R,7R,8R,14S)		1	4.23456667
88.9797408	100 - 29.1 - 6	http://nonlin	Canavalioside		1	0.73403333

92.4274284	100 - 36.4 - 7	http://nonlin	Morellinol			1	0.73403333
92.5375397	100 - 43.9 - 9	http://nonlin	Echinenone			1	0.73403333
92.5375397	100 - 43.9 - 9	http://nonlin	3-cis-Hydroxy-b,e-Caroten-			1	0.73403333
89.4877351	100 - 33.6 - 6	http://nonlin	Omadacycline			1	0.73403333
89.9941971	100 - 36.6 - 8	http://nonlin	Timosaponin A-I			1	4.20885
89.9941971	100 - 36.6 - 8	http://nonlin	(25S)-5beta-spirostan-3bet			1	0.73403333
93.4814245	100 - 36.6 - 7	http://nonlin	Cholesterol glucuronide			1	0.72545
93.9585324	100 - 35.4 - 7	http://nonlin	Tanespimycin			1	4.20885
90.4514488	100 - 43 - 9.8	http://nonlin	Caffeoylcycloartenol			1	0.73403333
88.6607506	100 - 44 - 9.8	http://nonlin	Lutein			1	0.73403333
82.8681954	100 - 36.6 - 8	http://nonlin	Agavoside A			1	0.73403333
91.2166982	100 - 37.7 - 8	http://nonlin	Tuberoside			1	4.20885
91.3090636	100 - 45.1 - 1	http://nonlin	Hydroxyspirilloxanthin			1	0.6826
91.3090636	100 - 45.1 - 1	http://nonlin	Spheroidenone			1	4.20885
91.3090636	100 - 45.1 - 1	http://nonlin	Thiothece-474			1	4.20885
92.6386759	100 - 33.5 - 7	http://nonlin	Polidocanol			1	4.23456667
94.8403235	100 - 35.7 - 8	http://nonlin	3,6-Anhydro-2,4,5-tris-O-(2			1	4.20885
87.3419065	100 - 36.5 - 7	http://nonlin	BQ-123			1	4.20885
75.8332918	100 - 35.6 - 8	http://nonlin	13-deoxytedanolide			1	4.20885
89.5406698	100 - 45.1 - 1	http://nonlin	(8'R)-Neochrome			1	0.73403333
94.624434	100 - 42.6 - 1	http://nonlin	Neferine			1	4.20885
89.6581447	100 - 39.9 - 9	http://nonlin	Momordicoside L			1	4.166
87.9131254	100 - 43.8 - 1	http://nonlin	Eldacimibe			1	4.20885
88.2047879	100 - 41.1 - 1	http://nonlin	Ginsenoside Rh5			1	4.20885
80.3186079	100 - 46.8 - 1	http://nonlin	Spirolide B			1	4.23456667
80.8446752	100 - 43.3 - 1	http://nonlin	trodusquemine			1	4.20885
82.578559	100 - 42.3 - 1	http://nonlin	MGDG(26:1/3:0)			1	0.6826

82.578559	100 - 42.3 - 1	http://nonlin	MGDG(3:0/26:1)		1	4.20885
88.616159	100 - 41.2 - 1	http://nonlin	Cyclopassifloside I		1	4.20885
88.616159	100 - 41.2 - 1	http://nonlin	Cyclopassifloside IV		1	4.20885
88.616159	100 - 41.2 - 1	http://nonlin	Cyclopassifloside X		1	4.20885
88.8013985	100 - 43.4 - 1	http://nonlin	MGDG(26:1/4:0)		1	4.20885
88.8013985	100 - 43.4 - 1	http://nonlin	MGDG(4:0/2	588.418433	1	0.73403333
96.1670933	100 - 45.3 - 1	http://nonlin	Myxochromide S2		1	4.20885
90.2935478	100 - 46.4 - 1	http://nonlin	Myxochromide S3		1	4.20885
89.1224781	100 - 54 - 15.	http://nonlin	Ubiquinone-10		1	4.20885
92.3867847	100 - 45.6 - 1	http://nonlin	Digitoxin		1	4.20885
92.3069691	100 - 43.4 - 1	http://nonlin	Alliosterol 1-rhamnoside 1		1	4.23456667
98.5031445	100 - 44.5 - 1	http://nonlin	1-PALMITOYL-2-OLEOYL-SN		1	4.20885
96.3303537	100 - 47.8 - 1	http://nonlin	Calenduloside G methyl es		1	4.20885
97.0932657	100 - 45.7 - 1	http://nonlin	Vinaginsenoside R11		1	4.20885
97.0932657	100 - 45.7 - 1	http://nonlin	Majonoside R2		1	4.20885
87.9193485	100 - 51.2 - 1	http://nonlin	Koryoginsenoside R1		1	4.20885
80.2049919	100 - 59.2 - 2	http://nonlin	Everolimus		1	4.20885
79.7733331	100 - 11 - 5.2	http://nonlin	Brassicanal A		1	5.08068333
93.7449593	100 - 5.71 - 0	http://nonlin	Guanidinoethyl methyl pho		1	4.47456667
87.5254842	100 - 18.6 - 3	http://nonlin	trans-3-Hydroxycotinine gl		1	0.69116667
90.2330145	100 - 19.4 - 3	http://nonlin	Tetraxetan		1	4.13171667
90.9197416	100 - 24.2 - 4	http://nonlin	Gentamycin (	463.298759	1	4.27743333
89.7373266	100 - 29.2 - 5	http://nonlin	Glycocholic acid		1	0.73403333
89.7373266	100 - 29.2 - 5	http://nonlin	3a,7b,12a-Trihydroxyoxoch		1	0.73403333
89.7373266	100 - 29.2 - 5	http://nonlin	Sodium glycocholate		1	0.73403333
91.8473904	100 - 33.1 - 6	http://nonlin	Pristimerin		1	4.14885
92.3307341	100 - 32.4 - 5	http://nonlin	Cervonyl carnitine		1	8.86495
80.5079976	100 - 19.2 - 4	http://nonlin	dTDP-3-amino-2,3,6-tridec		1	4.046

62.1461369	100 - 45 - 12.	http://nonlin	ERYTHROMYCIN PROPIONA		1	4.67458333
79.2566309	100 - 46.8 - 1	http://nonlin	1beta,2alpha,3beta,19alph		1	4.14885
81.2062812	100 - 66.6 - 2	http://nonlin	(3S)-3-{(2S,3R,5S)-5-[(2R)-3		1	4.14885

nin)
