# Supplementary material for: Metabolomic Profiles and Pathways in Osteoarthritic Human Cartilage: A Comparative Analysis with Healthy Cartilage
Source: Metabolites. 2024 Mar 25;14(4):183. doi: 10.3390/metabo14040183 (PMC11051929; doi:10.3390/metabo14040183)
Supplement: Supplementary file 1 [file metabolites-14-00183-s001.zip › HealthyvsDiseased_SuppFiguresTables.pdf]

SUPPLEMENTAL FIGURES

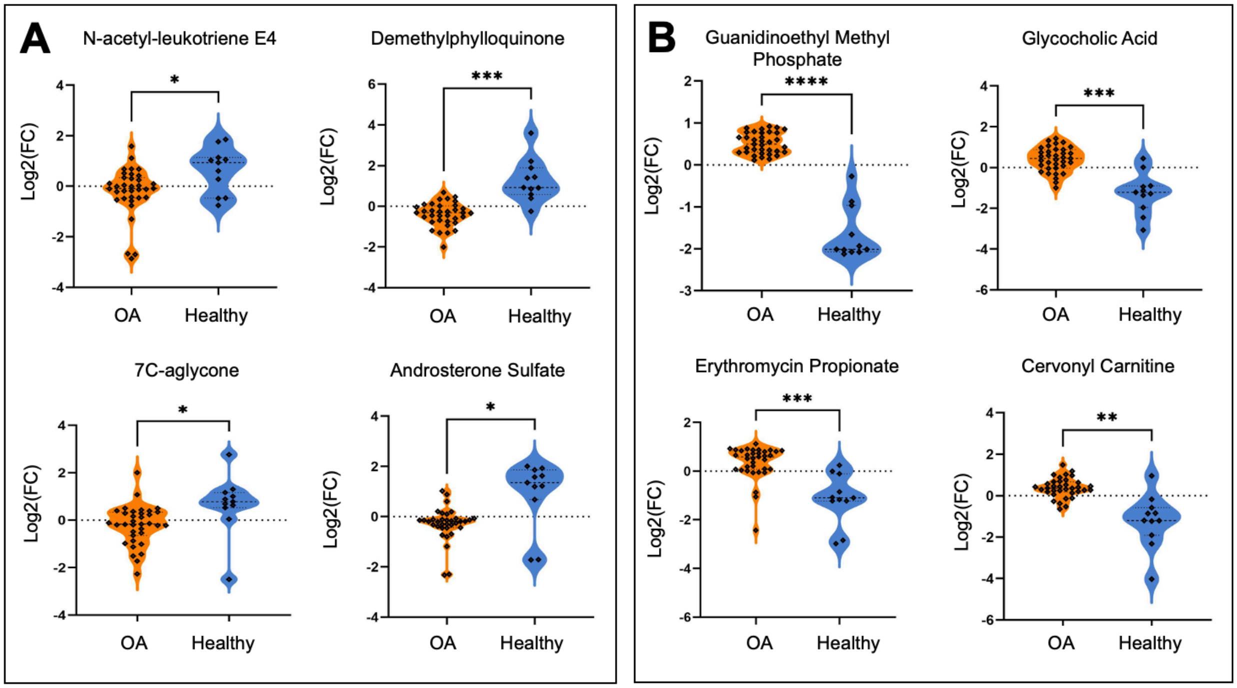

Supplemental Figure 1. Identified metabolites differ in abundance between healthy and osteoarthritic cartilage. (A) Metabolites – N-acetyl-leukotriene E4, demethylphylloquinone, 7C-aglycone, androsterone sulfate – are higher in abundance in healthy cartilage compared to osteoarthritic cartilage. (B) Conversely, metabolites - guanidinoethyl methyl phosphate, glycocholic acid, erythromycin propionate, cervonyl carnitine – are higher in abundance in cartilage from individuals with osteoarthritis compared to healthy cartilage. Mass-to-charge intensities of interest were normalized and used to generate plots. To correct for multiple comparisons, FDR p-value corrections were performed and were less than < 0.05. Moreover, Welch's t-tests were performed for each identified metabolite. Orange = osteoarthritis. Blue = healthy. **** = p < 0.0001, *** p < 0.0002, ** p < 0.001, * = p < 0.05.

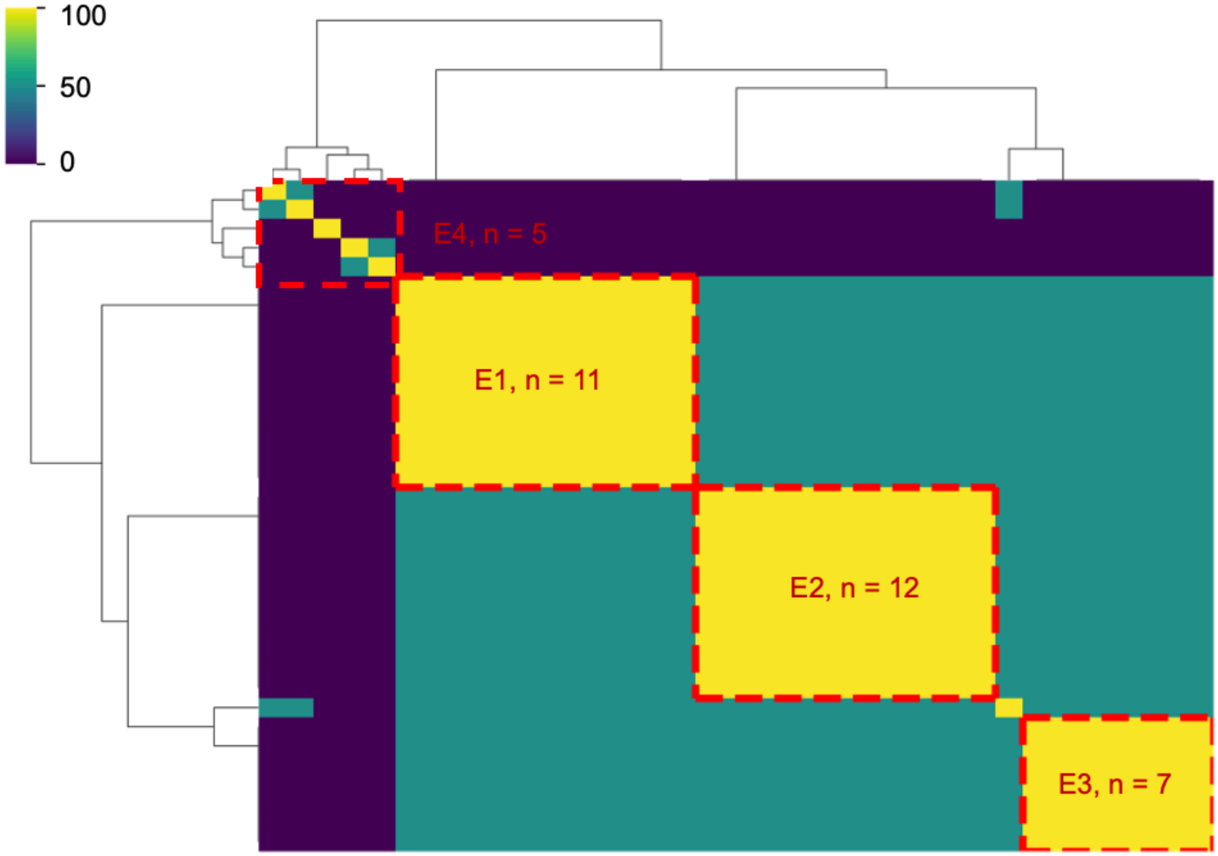

Supplemental Figure 2. Ensemble clustering of osteoarthritis participants revealed distinct metabolic endotypes. By employing ensemble clustering of all osteoarthritis participants based on metabolomic data, four unique endotypes were revealed. Specifically, endotype one (E1) is composed of n = 11 participants. Moreover, endotype two (E2), three (E3), and four (E4) are composed of n = 12, n = 7, and n = 5, respectively. Scale bar represents the percentage (%) of time participants cluster together.

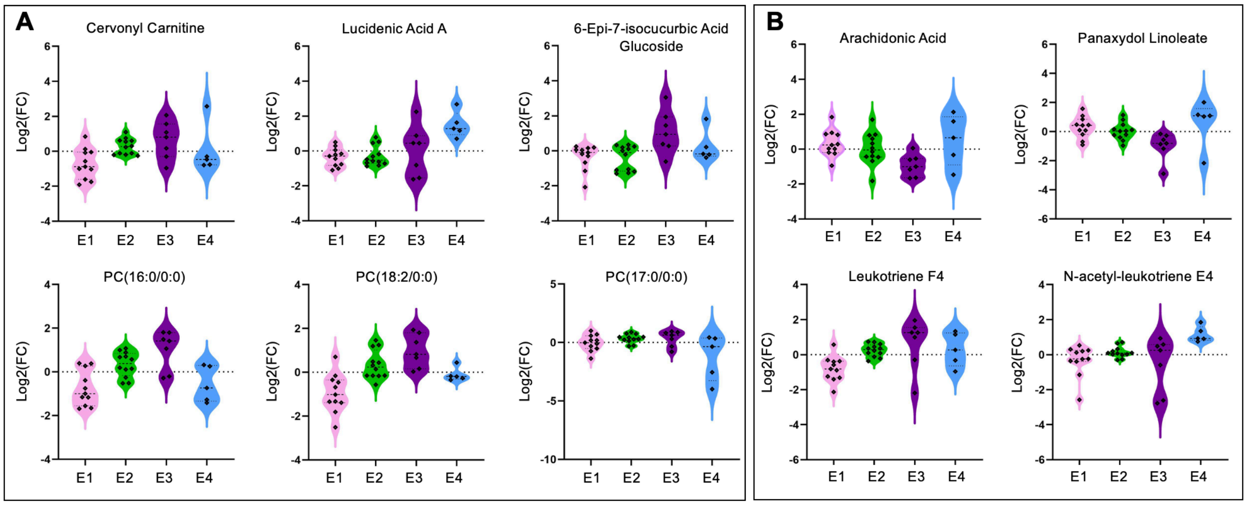

Supplemental Figure 3. Identified metabolites differ in abundance across osteoarthritis endotypes. (A) Lipid and lipid-like identified metabolites that are differentially regulated across osteoarthritis endotypes. (B) Identified metabolites that differ in abundance across endotypes and are associated with arachidonic acid metabolism and leukotriene metabolism. Mass-to-charge intensities of interest were normalized and used to generate plots. To correct for multiple comparisons, FDR p-value corrections were performed and were less than < 0.05. Moreover, Welch's t-tests were performed for each identified metabolite. Endotype colors correspond to: pink – Endotype 1; green – Endotype 2; purple – Endotype 3; blue – Endotype 4.

SUPPLEMENTAL TABLE CAPTIONS

Supplemental Table 1. Participant information including donor number, assigned anonymous identifier, age, sex, and osteoarthritis endotype group.

Supplemental Table 2. All metabolic pathways determined from MetaboAnalyst when comparing healthy and diseased cartilage using volcano plot analyses. Populations of metabolite features are defined on Figure 1C.

Supplemental Table 3. Putatively identified metabolites that differ in abundance between healthy and diseased cartilage distinguished by volcano plot analysis. Identifications were made by performing liquid chromatography tandem mass spectrometry (LC-MS/MS). For all identified, information includes observed and theoretical mass-to-charge ratios, parts per million (ppm) error, accepted compound ID and description, adduct, chemical formula, total score out of 100, and fragmentation score. Identifications with error greater than 20 ppm, total score < 60, and a fragmentation score < 12 were excluded.

Supplemental Table 4. Putatively identified metabolites that differ in abundance between healthy and diseased cartilage distinguished by t-test analysis. Identifications were made by performing liquid chromatography tandem mass spectrometry (LC-MS/MS). For all identified, information includes observed and theoretical mass-to-charge ratios, parts per million (ppm) error, accepted compound ID and description, adduct, chemical formula, total score out of 100, and fragmentation score. Identifications with error greater than 20 ppm, total score < 60, and a fragmentation score < 12 were excluded.

Supplemental Table 5. Putatively identified metabolites that differ in abundance between osteoarthritis endotype groups distinguished by ANOVA analysis. Identifications were made by performing liquid chromatography tandem mass spectrometry (LC-MS/MS). For all identified, information includes observed and theoretical mass-to-charge ratios, parts per million (ppm) error, accepted compound ID and description, adduct, chemical formula, total score out of 100, and fragmentation score. Identifications with error greater than 20 ppm, total score < 60, and a fragmentation score < 12 were excluded. All metabolite features with an FDR-corrected p-value > 0.05 distinguished by ANOVA when comparing all four osteoarthritis groups.

Supplemental Table 6. All metabolic pathways determined from MetaboAnalyst when comparing osteoarthritis endotypes using median metabolite intensity heatmap analysis. Clusters defined on Figure 2E.

Supplementary Table 7. Raw data from XCMS.
